# Active TB case finding in returnee coalminers and their home district communities in Pakistan

**DOI:** 10.5588/pha.25.0031

**Published:** 2025-12-03

**Authors:** K.U. Eman, G.N. Kazi, U.R. Lodhi, B. Kirubi, M. Ali, M. Shahzad, F. Siraj, S.A. Raisani, S. Ashraf, S. John, S. Tahseen, J. Creswell

**Affiliations:** 1 Dopasi Foundation, Islamabad, Pakistan;; 2 Stop TB Partnership, Geneva, Switzerland;; 3 Provincial TB Control Program, Peshawar, Pakistan;; 4 Provincial TB Control Program, Quetta, Pakistan;; 5 Communicable Disease Control, Hyderabad, Pakistan;; 6 Janna Health Foundation, Yola, Nigeria.

**Keywords:** tuberculosis, ultra-portable X-ray, AI-enabled CXR, vulnerable population, chest X-ray, TB screening

## Abstract

**SETTING:**

Five districts with large coalmining workforces in Pakistan.

**OBJECTIVE:**

To assess the burden of TB among returnee coalminers (RCMs) and associated community members (ACMs) in their home districts through active case finding (ACF).

**DESIGN:**

This cross-sectional study (October 2020–September 2021) used portable chest X-ray (CXR) with artificial intelligence (AI) in camps and verbal screening in camps and communities. Individuals screening positive were tested with GeneXpert, and those diagnosed were initiated on TB treatment.

**RESULTS:**

A total of 150,242 individuals were screened, including 44% RCMs. Of these, 8.3% underwent CXR, 10% verbal screening in camps, and 81% verbal screening in communities. Symptoms were reported by 45% of RCMs and 15% of ACMs, while CXR abnormalities were comparable. TB was diagnosed in 226 RCMs and 204 ACMs, with overall prevalence per 100,000 of 341 (95% confidence interval [CI]: 296–385) and 243 (95% CI: 209–276), respectively. TB prevalence varied by screening strategy and was significantly higher among those screened with CXR: 1,156 in RCMs and 497 in ACMs.

**CONCLUSION:**

AI-assisted CXR was substantially more effective than verbal screening, detecting significantly higher numbers of TB cases among RCMs and ACMs, supporting its use for targeted ACF in high-risk populations.

Reaching people with TB that are ‘missed’ by the health system remains a major public health challenge.^1,2^ Globally, around 11 million people develop active TB each year,^3^ a fourth of which (4.3 million) remain unreported to national TB programmes,^4,5^ leading to prolonged suffering and ongoing transmission.^6^ Socio-economic, cultural, and health-related risk factors disproportionately affect specific populations, rendering them socially invisible with restricted health care access. These populations include the homeless, people with HIV, migrants, refugees, children, urban slum residents, and miners.^7^ The WHO End TB Strategy and Stop TB Partnership (STP) Global Plan to End TB emphasise diagnosis and successful treatment of at least 90% of people with targeted approaches to find TB in key and vulnerable populations, who would otherwise remain undetected.^7–9^ Coalminers are particularly vulnerable due to their occupational exposure to coal dust compromising their lung function and increasing TB risk.^10^ South Africa has reported the world’s highest rates of TB among mine workers,^11^ with incidence reported up to 10 times more than the general population.^12^ Furthermore, many miners are migrants, which may also facilitate TB transmission to general communities.^13^ When ill, miners often return home making their families and rural communities particularly vulnerable due to their circular patterns of traveling between their mines and homes.^14^

Pakistan, a high-TB-burden country, predominantly relies on passive case finding among people with TB symptoms seeking care at health care facilities. Despite being a key contributor to national economies, the coalmining sector lacks robust application of occupational health and safety regulations, exposing unprotected workers to significant hazards.^15^ As in South Africa, the circular migration pattern also prevails in Pakistan. Coalmine workers migrate from various districts in Pakistan and even neighbouring Afghanistan, to the coalmining districts for their livelihood. However, upon falling sick, they often return to their home districts, usually in remote areas with limited access to health care, carrying a high transmission risk to broader communities. An earlier intervention of ours, supported by the STP, TB REACH Wave 6, was implemented in Pakistan for active case finding (ACF) among coalminers and their associated communities at five mining sites of Balochistan.^16^ Our results showed no significant difference in TB prevalence between miners, their associated communities, and the general population, in sharp contrast to published literature suggesting a substantially increased risk of TB among miners.^16^ A plausible explanation for under-detection of the true TB burden in our study is the targeted approach used for screening active miners, which likely excluded workers who had returned to their home districts due to illness or disability. Furthermore, reliance on symptom-based screening may have missed individuals with asymptomatic TB.

To explore this further, we designed an intervention to determine TB prevalence among returnee coalminers (RCMs) and associated communities within their home districts using ACF.

## METHODS

This cross-sectional study was conducted over a 12-month period, from 6^th^ October 2020 to 3^rd^ September 2021. We recruited individuals aged 18 years and above residing at study sites and were defined as 1) RCMs having an occupational history of working at coalmines and 2) associated community members (ACMs) for the entire community residing in the selected intervention sites.

### Selection of intervention sites

**FIGURE 1.**
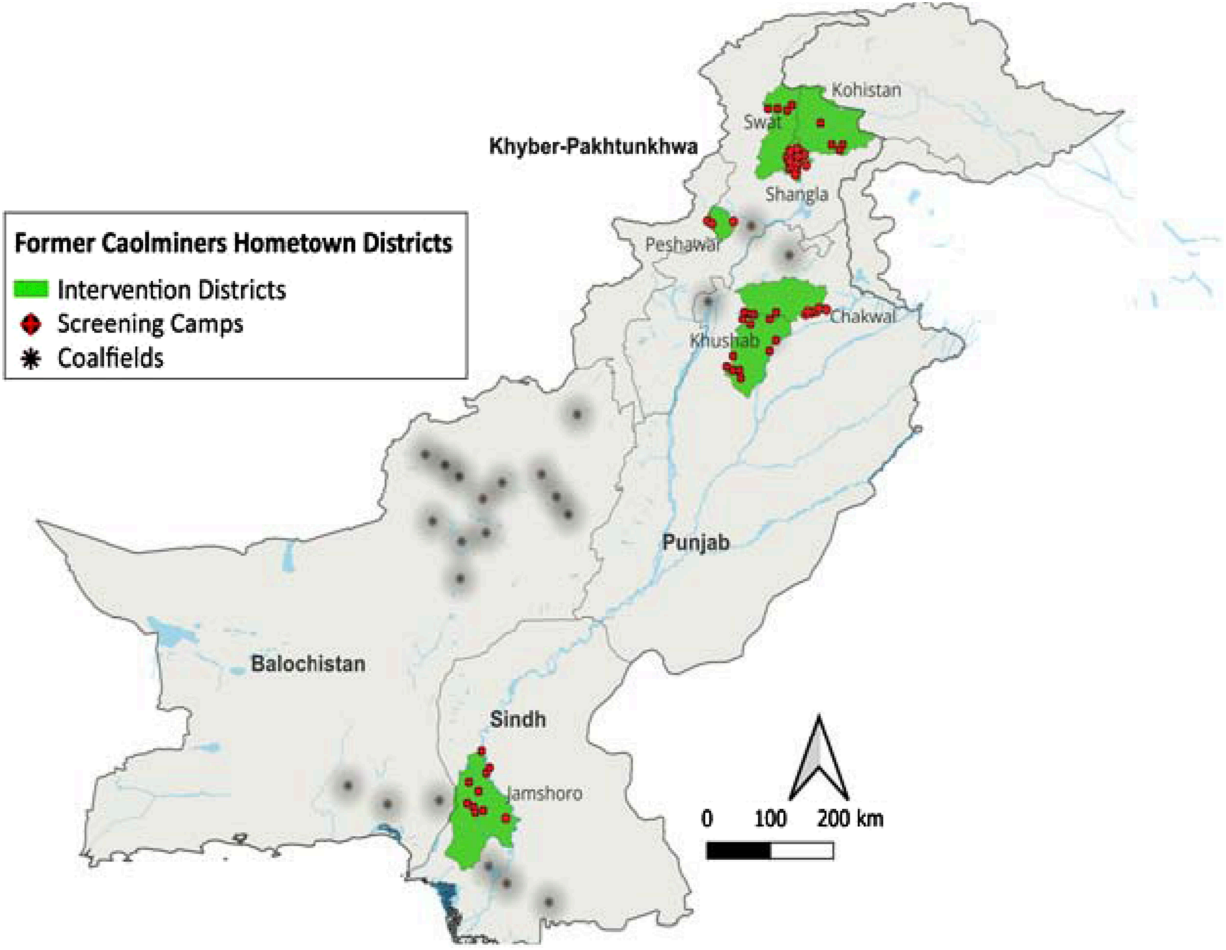
Geographical distribution of coalmines and the home districts of coalminers, as intervention districts for the study.

A list of major registered coalminers was obtained from the relevant departments^10^ and a mapping exercise was conducted. Five districts across three provinces (Khyber Pakhtunkhwa, Punjab, and Sindh) with large coalmining workforces were identified, and within these 50 union councils (UCs, the smallest rural administrative units) were selected for this intervention (Figure 1).

### The Intervention

The ACF intervention involved four strategies:

1. For the first time in Pakistan, chest X-ray (CXR) screening camps utilised ultra-portable, lightweight, battery-powered Fujifilm Xair X-ray devices (Fujifilm Corporation, Tokyo, Japan), which were equipped with artificial intelligence (AI) software Lunit Insight CXR (Lunit Inc., Seoul, Republic of Korea). A trained three-member team, comprising of a counsellor, data collector, and X-ray technician conducted the CXR screening camps around village centres or houses of the community elders. Prior community mobilisation encouraged participation. Camps were held on alternate days, aiming to screen 100 people per day at sites spaced around 2 kilometres apart.
2. Verbal symptoms screening camps were organised to increase the ACF reach and facilitate individuals unable to access CXR camps. The WHO four-symptoms-based screening criteria, encompassing cough, fever, weight loss, and night sweats, were used to identify TB presumptives.^17,18^
3. Door-to-door screening in community outreach was carried out to facilitate household members, particularly women, having difficulty in attending the camps. Ten teams, each comprising three to four individuals, including a designated field officer, data collector, and two locally hired paramedics, worked under the respective district coordinators.
4. Screening of contacts was conducted after securing household details from the newly diagnosed bacteriologically confirmed TB patient. Field staff visited their homes; contacts were verbally screened and sputum collected if symptomatic.

### Laboratory testing

Sputum samples were collected on-site from individuals identified as presumptive for TB based on the CXR score (>0.5 on Lunit Insight) or TB symptoms. Sputum samples were transported to the nearest pre-identified public sector health facility for Xpert MTB/RIF testing. Individuals unable to provide a sputum sample underwent clinical evaluation. Those diagnosed with TB were enrolled for treatment at the respective public sector health facility.

### Data collection and analysis

Data, including demographics, occupational history, screening and laboratory test results, clinical diagnoses, and TB treatment initiation and outcomes, were collected on paper-forms and entered on a Microsoft Excel database. Descriptive statistics included frequencies and percentages calculated for the TB screening cascade from screening to treatment completion. The number needed to screen (NNS) and number needed to test (NNT) to find one bacteriologically confirmed TB case were determined. TB prevalence per 100,000 was calculated for the entire screened population and analysed by screening methodology, with comparisons made between RCMs and ACMs. As the study was conducted during the peak of the COVID-19 pandemic, implementation was partially disrupted, limiting both ACF activities and comprehensive data collection.

### Ethical statement

All activities were implemented in line with the National TB Guidelines for ACF among high-risk populations and approved by the National and Provincial TB Control Programs. As this was a routine programmatic intervention, separate IRB approval was not required. Participation was voluntary, verbal informed consent was obtained, and all data were anonymised to ensure confidentiality and privacy.

## RESULTS

**FIGURE 2.**
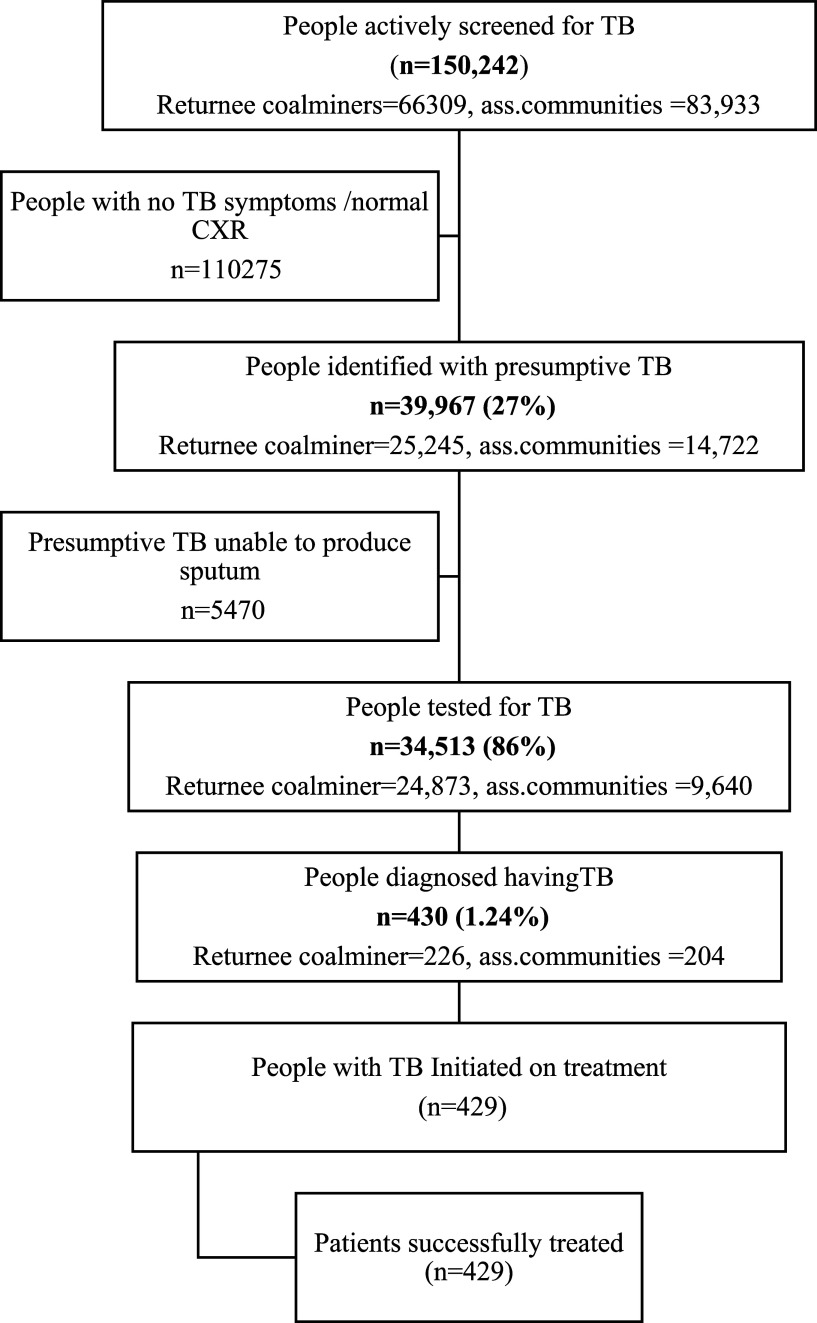
Flow chart presenting participant enrolment and sequential steps in TB care pathway.

A total of 150,242 individuals were screened, including 66,309 (44%) RCMs and 83,933 (66%) ACMs in five intervention districts (Figure 2). Of the total population screened, 8.3% were screened in 117 CXR screening camps, 10% in 105 verbal symptoms screening camps, and 80% through door-to-door outreach using symptoms screening (Table 1). Household contacts of TB patients comprised 0.7% (1,031) of the screened population. The summary results of individuals screened using different screening approaches, NNS, NNT, and the TB prevalence estimates, are given in Table 2.

**TABLE 1.** Intervention districts, interventions, population screened, and TB case diagnosed.

Intervention area			Estimated population of returnee coalminers	Chest camps	People screened			TB cases diagnosed
Province	District	Village union councils			Returnee coalminers	Associated community members	Total	
KP	Shangla	23	69,500	93	45,173	44,164	89,337	271
	Swat	2	3,500	5	831	2,512	3,343	9
Punjab	Chakwal	13	11,700	69	8,634	16,562	25,196	65
	Khushab	8	9,500	46	6,611	11,950	18,561	48
Sindh	Jamshoro	4	12,500	9	5,060	8,745	13,805	37
Total		50	106,700	222	66,309	83,933	150,242	430

**TABLE 2.** Screening and diagnostic yield of different approaches in TB care cascade.

Screening methodology and outcome	All	Returnee coalminers	Associated communities^**A**^	*P* value
People screened in camps with chest X-ray	12,495	6,662	5,833	
People screened positive (TB presumptive)	1,288 (10%)	731 (11%)	557 (10%)	0.009
Among screened positive, people able to produce sputum and tested	833 (64.7%)	470 (64.3%)	363 (65.2%)	0.744
Laboratory-confirmed TB cases diagnosed	106 (12.7%)	77 (16.4%)	29 (8.0%)	<0.001
Number needed to screen to find one TB case (NNS)	117.9	86.5	201.1	
Number needed to test to detect one TB case (NNT)	7.9	6.1	12.5	
Estimated prevalence of TB in 100,000	848	1,156	497	
People screened in camps using symptom screening	14,965	8,125	6,840	
People screened positive (TB presumptive)	6,565 (44%)	4,436 (55%)	2,129 (31%)	<0.001
Among screened positive, people able to produce sputum and tested	6,240 (95%)	4,325 (97%)	1,915 (90%)	<0.001
Laboratory-confirmed TB cases diagnosed among tested	97 (1.6%)	46 (1.1%)	51 (2.7%)	<0.001
Number needed to screen to find one TB case (NNS)	154.3	176.6	134.1	
Number needed to test to detect one TB case (NNT)	64.3	94.0	37.5	
Estimated prevalence of TB in 100,000	648	566	746	
Door-to-door symptom screening in community outreach	121,751	51,522	70,229	
People screened positive (TB presumptive)	32,065 (26.3%)	22,521 (43.7%)	9,544 (13.6%)	<0.001
Among screened positive, people able to produce sputum and tested	27,391 (85.4%)	20,078 (89.2%)	7,313 (76.6%)	<0.001
Laboratory-confirmed TB cases diagnosed among tested	184 (0.7%)	91 (0.5%)	93 (1.3%)	<0.001
Number needed to screen to find one TB case (NNS)	661.7	566.2	755.2	
Number needed to test to detect one TB case (NNT)	148.9	220.6	78.6	
Estimated prevalence of TB in 100,000	151	177	132	

A Contact screening (not shown): 1,031 household contacts screened; among these, 49 screened positive and 33 were able to expectorate; 3 TB cases diagnosed with corresponding NNS of 344, NNT of 11, and estimated TB prevalence of 291.

Among 12,495 people screened by CXR, 10% had CXR findings consistent with TB and 65% of these were able to expectorate with no difference in proportion between RCMs and ACMs (Table 2). Among the 136,716 individuals verbally screened, 28% reported symptoms, with a three-fold higher frequency among RCMs (45%) compared with ACMs (15%). Of those with symptoms, 87% were able to expectorate sputum, with a significantly higher proportion among RCMs than ACMs.

The estimated TB prevalence was highest among RCMs screened by CXR (1,156 per 100,000), corresponding to 11% screening positive and the lowest NNS (86) and NNT (6.1). Among ACMs, highest estimates were reported among the group screened by symptoms in camps (746), with 31% screen positive and lowest NNS (134), while NNT (12.5%) was lower in group screened by CXR (Table 2). Among the total population screened, 114,593 (76.3%) were male, which included all returnee coalminers (66,309) and 58% (48,283) of the ACMs (Table 3). The proportion of TB presumptives was significantly higher among RCMs (38%) than among men (15%) or women (21%) from ACMs (Table 3). A total of 390 bacteriologically confirmed persons with TB were diagnosed, including 214 in RCMs and 176 in ACM groups. In addition, 40 persons with TB were clinically diagnosed, 12 in RCMs and 28 in ACMs. The proportion of bacteriologically confirmed TB was 90.6% among all TB cases detected, 95.0% among RCMs and 86.0% among ACMs.

**TABLE 3.** Prevalence of TB among population screened by gender.

Characteristics	Cumulative	Returnee coalminers (all men)	Associated community		
			Total	Men	Women
Total Screened	150,242	66,309	83,933	48,283	35,650
Screened positive (TB Presumptive)	39,967	25,245	14,722	7,111	7,611
	26.6%	38.1%	17.5%	14.7%	21.3%
Laboratory-confirmed TB cases diagnosed	390	214	176	87	89
Total TB cases diagnosed (laboratory confirmed and clinically diagnosed)	430	226	204	100	104
Positivity ratio (TB diagnosed case/Total screened)	0.29%	0.34%	0.24%	0.21%	0.29%
Crude prevalence/100,000 population	286 (259–313)	341 (296, 385)	243 (209, 276)	207 (166, 247)	292 (236, 347)
Crude prevalence ratio (PR)	—	1.40 (1.16, 1.69)	Ref.	Ref.	1.4 (1.07, 1.85)
*P* value	—	0.0004			0.0143

The overall crude estimate of TB prevalence was 286/100,000 population (95% confidence interval [CI]: 259–313). TB prevalence among RCMs was 341 (95% CI: 296–385), compared with 243 (95% CI: 209–276) in the ACMs. Among ACMs, the estimated TB prevalence was higher among women as compared with men (Table 3).

## DISCUSSION

To our knowledge, this was the first intervention of its type in Pakistan, and globally, to deploy ultra-portable X-ray devices equipped with AI for TB screening. The estimated overall prevalence of PTB was 341/100,000 in RCMs and 243/100,000 in ACMs, lower than the national estimate of 397/100,000.^19^ However, TB prevalence varied substantially by the screening strategy. Among RCMs and ACMs screened in CXR camps, TB prevalence compared with the overall estimate was more than three times higher in RCMs (1,156 vs. 341) and two times higher in ACMs (496 vs. 243).

While TB prevalence among RCMs in our study was lower than that reported in South African gold miners, likely due to lower HIV prevalence and differences in the extracted mineral.^3^ However, our findings align with studies from Myanmar, where ACF in remote mining areas revealed higher TB prevalence among miners compared with the general population,^20^ and from India, which reported a 1.6-fold higher prevalence of TB and silico-TB among former coalminers relative to non-miners following prolonged silica dust exposure.^21^ Compared with our previous study at coal mining sites,^16^ the overall TB prevalence was 1.5 times higher in RCMs (341/100,000 vs. 233/100,000) and only marginally high in ACMs (243/100,000 vs. 234/100,000).^16^ However, TB prevalence in both RCMs and ACMs was particularly elevated in subset of population screened in CXR camps (1,156/100,000 and 496/100,000) and in verbal screening camps (566/100,000 and 746/100,000) but not in community outreach (175/100,000 and 132/100,000). The findings highlight the hidden burden that is missed when relying solely on symptom-based screening. Elevated prevalence in ACMs underscores the potential for transmission from RCMs to surrounding communities. Our findings also suggest a higher TB burden among RCMs compared with our previous study on active miners,^16^ as a higher TB case detection was observed in the subset of RCM population screened using the same verbal screening strategy in chest camps. These findings underscore the importance of community-based screening for returnee miners and their households to better capture the hidden burden of TB.

To evaluate the efficiency of various screening approaches, we compared the proportion of presumptives identified, NNS and NNT across different population subsets and ACF strategies. Among those RCMs screened in CXR camps, the proportion of screens that were positive, and NNS and NNT were 11%, 86.5 and 6.1 respectively, compared with 55%, 176 and 94 for symptom screening, indicating that CXR is a more efficient tool for screening and case finding among RCMs. These findings are consistent with the results of a systematic review, which reported that in miners, the weighted mean NNS using CXR alone was 66 (range: 29–100), while NNS for a positive symptoms’ screening alone was 207.^22^ However, in contrast, among ACMs the NNS was lowest in subset of population screened by symptoms-based camps suggesting that symptom-based screening work effectively in populations without associated lung conditions. Our findings further support that the NNS is inversely correlated with TB prevalence, consistent with evidence from previous systematic reviews.^8^ This reinforces that ACF strategies are more efficient and yield greater returns when focused on populations with elevated TB risk.

Exposure to coalmine dust causes a high burden of coal mine dust lung diseases.^23^ In our study, 45% of RCMs reported symptoms three times higher than ACMs and four times higher than the general population.^19^ The high prevalence of respiratory symptoms among coalminers also suggests that symptom-based TB screening is not a particularly efficient strategy for coalminers.^6^ Our findings corroborate evidence that CXR outperforms symptom-based screening, with higher sensitivity, specificity, and stronger correlation with sputum culture.^8,24^ CXR screening detects more cases than verbal screening and is also more cost-effective, consistent with reports from Uganda,^24^ Vietnam,^25^ and Zimbabwe.^26^ These results highlight the importance of integrating AI-assisted CXR into ACF strategies, particularly for high-risk populations.

Differences in the proportions of TB cases detected between RCMs and ACMs across screening approaches in our study likely reflect underlying health status and variations in health-seeking behaviour. Highly symptomatic miners were more inclined to attend CXR camps, whereas ACMs, particularly women, opted for the more accessible symptom-based camps, explaining the higher proportion of TB cases detected among female community members. A recent Nigerian study demonstrated that nomadic populations with very high rates of symptoms had lower-than-expected additional yield from CXR. However, both the verbal screening camps and CXR camps detected much more TB cases than door-to-door screening.^19^ In our study, door-to-door symptom screening covered 81% (121,751) of the study population and identified 47% (184/390) of all TB cases. This approach diagnosed 43% (91/214) of reported TB cases in the RCMs and 54% in the ACMs despite having a higher NNS and NNT. Although TB prevalence detected through this approach is below the WHO threshold for community ACF,^17^ it still underscores the presence of undiagnosed or ‘missed’ TB cases within the community. It also provides valuable insight into the health-seeking behaviour and lack of motivation in substantial proportion of target vulnerable populations with respiratory symptoms to attend community-based screening camps for TB. Coalminers in particular may have limited interest to seek TB screening alone, as it fails to address their broader respiratory health concerns. This underscores the need for integrated TB and lung health services tailored to the population’s needs.^13^

For contact investigation, although it comprised less than 1% of the our total study population, the yield was 11% (3/33) among screen positive contacts tested, consistent with the high yield documented in other studies.^20^ Furthermore our intervention demonstrated excellent treatment success rates (over 99.7% cured), effectively overcoming barriers faced by RCMs in remote areas, who are often otherwise missed by routine passive case-finding approaches.^27^ Deployment of mobile teams to hard-to-reach mining communities has been shown to be an effective strategy for identifying people with TB by others.^20,28^ Analyses of the cost-effectiveness and trade-offs of the different screening strategies are warranted.

This study has limitations. COVID-19 restrictions prevented collection of detailed occupational histories, and only one screening method was applied per participant, precluding direct comparison of CXR and verbal screening. Limited CXR availability also meant few participants were screened with this method, likely leading to under-detection of TB cases.

## CONCLUSION

This study shows a higher TB prevalence among RCMs, with evidence of transmission to nearby communities. Ultra-portable CXR with AI improved case detection over verbal screening, underscoring the value of targeted ACF with innovative diagnostics for high-risk groups and their communities.
